# Impact of isotretinoin therapy on thyroid hormone levels in acne vulgaris: A prospective study

**DOI:** 10.1097/MD.0000000000044236

**Published:** 2025-08-29

**Authors:** Mamdouh Ali Kotb, Salman Bin Dayel, Othman Abahussein, Ramadan S. Hussein

**Affiliations:** a Department of Internal Medicine, College of Medicine, Prince Sattam Bin Abdulaziz University, Al-Kharj, Saudi Arabia; b Department of Dermatology, College of Medicine, Prince Sattam Bin Abdulaziz University, Al-Kharj, Saudi Arabia.

**Keywords:** acne vulgaris, isotretinoin, prospective study, thyroid function, thyroid hormones

## Abstract

Acne vulgaris (AV) is a common dermatological condition characterized by inflammatory and noninflammatory skin lesions. Isotretinoin (IOS), a systemic retinoid, is widely recognized for its efficacy in managing severe AV due to its potent anti-inflammatory and sebosuppressive properties. IOS interacts with nuclear receptors belonging to the steroid-thyroid hormone superfamily. While its therapeutic benefits are well-documented, the potential effects of IOS on thyroid function remain inadequately explored. This study aims to evaluate the impact of IOS therapy on thyroid hormone levels in patients with AV. This prospective study included 50 patients with AV who were prescribed 0.5 to 1 mg/kg/day of IOS treatment for 4 months. Thyroid function was evaluated by measuring serum levels of triiodothyronine, thyroid-stimulating hormone, and thyroxine at the beginning and post-IOS treatment period. Posttreatment analyses revealed a significant increase in thyroid-stimulating hormone levels and a substantial decrease in triiodothyronine and thyroxine levels compared to baseline measurements (*P* < .000). These findings suggest that IOS therapy may influence thyroid hormone synthesis and regulation. IOS may induce subclinical changes in thyroid function. Monitoring thyroid parameters during therapy is recommended. Further research with larger sample sizes and controlled designs is warranted to better understand the mechanisms underlying these associations and their clinical implications.

## 1. Introduction

Acne vulgaris (AV) is a chronic inflammatory condition that predominantly affects adolescents and young adults, peaking during puberty. It presents with both inflammatory lesions (papules, pustules) and noninflammatory lesions (comedones), typically on sebaceous-rich areas such as the face, chest, and back. The psychosocial impact of AV is well-documented, often necessitating timely and effective intervention.^[1–4]^

Isotretinoin (IOS), a systemic retinoid, remains the gold standard for treating severe or recalcitrant AV due to its ability to reduce sebaceous gland size, suppress sebum production, and modulate inflammation.^[5–7]^ While IOS’s dermatologic efficacy is established, its broad systemic activity raises concerns about potential effects on other organ systems, including endocrine pathways.^[8–12]^

Among the endocrine concerns, alterations in thyroid hormone profiles during IOS therapy have been inconsistently reported. Some animal and human studies suggest IOS may influence thyroid-stimulating hormone (TSH), triiodothyronine (T3), and thyroxine (T4) levels, while others do not.^[13,14]^ Given IOS’s interaction with nuclear receptors from the steroid-thyroid hormone receptor family, these findings warrant further exploration.

This prospective study was designed to evaluate the impact of systemic IOS therapy on thyroid hormone levels (T3, T4, and TSH) in patients with AV. By using paired pre- and posttreatment measurements in a real-world clinical setting, we aimed to clarify whether clinically meaningful changes occur and to inform appropriate monitoring strategies.

## 2. Materials and methods

### 2.1. Study design

This prospective study was conducted at a dermatology outpatient clinic from February 2024 to May 2024. While the study did not include a control group, efforts were made to minimize potential biases through stringent inclusion and exclusion criteria. The absence of control measures is acknowledged as a limitation, and future studies are recommended to include a comparison cohort.

### 2.2. Participants

Fifty AV patients (age 18–35) undergoing systemic IOS therapy and meeting inclusion/exclusion criteria were enrolled.

#### 2.2.1. Inclusion criteria

1. Diagnosis of AV confirmed using the Global Acne Grading System.
2. Minimum duration of systemic IOS treatment: 4 months.
3. Age range: 18 to 35 years.
4. Both male participants and single females were included.
5. All participants who met the eligibility criteria gave written informed consent.

#### 2.2.2. Exclusion criteria

1. Individuals with preexisting thyroid disorders, including hypothyroidism or hyperthyroidism.
2. History of kidney or liver dysfunction.
3. Presence of systemic diseases influencing thyroid function.
4. Contraindications to systemic IOS treatment, such as pregnancy, lactation, or advanced age.
5. Presence of severe dermatological conditions unrelated to AV that could interfere with study outcomes.
6. Inability to comply with the study protocol or attend follow-up visits.

The exclusion of married women was based on the need to control for potential confounding hormonal factors. This limitation and its implications for generalizability are acknowledged in the discussion.

### 2.3. Intervention

Each participant underwent a comprehensive evaluation process, including detailed medical history, physical examination, and acne severity assessment using Global Acne Grading System. IOS therapy was initiated at a dosage of 0.5 to 1 mg/kg body weight, administered twice daily with meals, and continued for a minimum of 4 months. Adherence to treatment was monitored through patient diaries and regular follow-up visits. Participants were observed for side effects throughout the treatment period.

### 2.4. Data collection

Biochemical parameters, including serum levels of T3, T4, and TSH, were assessed before and 4 months after initiating IOS therapy. Fasting blood samples were collected (timing not standardized [see Limitations]) via venepuncture following a 12-hour fasting period, immediately centrifuged to separate plasma, and analyzed using electrochemiluminescent immunoassay methods. Normal ranges were defined as follows: TSH (0.30–4.20 μIU/mL), T4 (12.0–22.0 pmol/L), and T3 (3.10–6.80 pmol/L). The choice of a 4-month follow-up period was based on the typical duration of IOS therapy; however, potential delayed hormonal effects warrant further investigation in future studies.

### 2.5. Statistical analysis

Statistical analyses were performed using the IBM Statistical Package for the Social Sciences for Windows, version 26 (IBM Corp., Armonk). Normality of data distribution was assessed using the Kolmogorov–Smirnov test. Descriptive statistics were calculated for mean, standard deviation (SD), and percentage values. A paired sample *t* test was employed to compare pre- and posttreatment thyroid hormone levels. Regression analyses were considered to adjust for potential confounders such as age, sex, and baseline hormone levels. Statistical significance was defined as *P* < .05.

### 2.6. Ethical considerations

The study followed the Declaration of Helsinki ethical guidelines, with all participants providing written informed consent, and their privacy and confidentiality were rigorously protected. The research protocol received approval from the institutional review board at Prince Sattam Bin Abdulaziz University (Approval No. SCBR-213/2024).

### 2.7. Sample size determination

**Figure 1.**
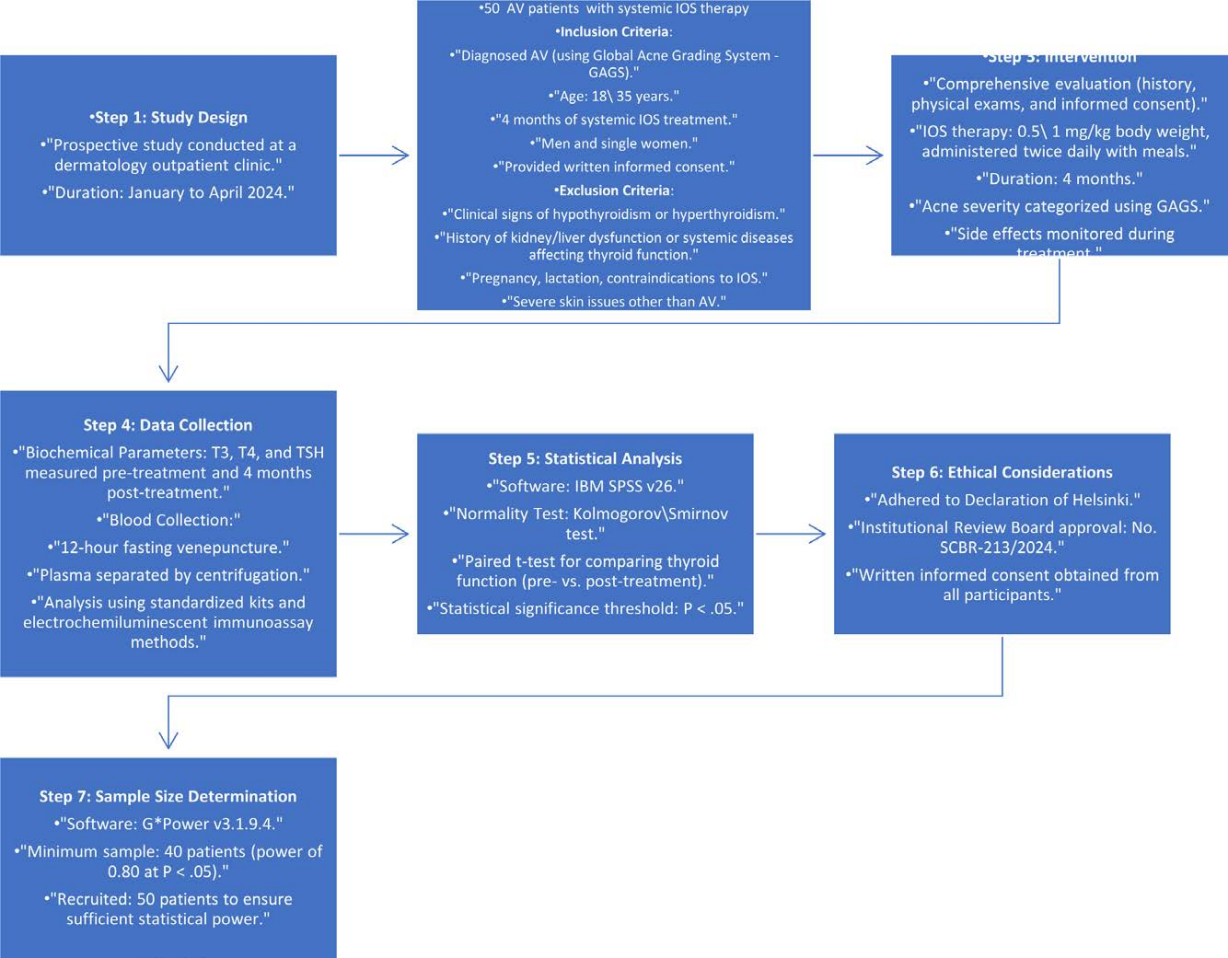
Materials and methods flowchart.

The minimum required sample size was calculated using G*Power software version 3.1.9.4 (Heinrich-Heine-Universität Düsseldorf, Düsseldorf, Germany), based on data from prior studies. A sample size of 40 participants was estimated to detect significant changes in serum T3, T4, and TSH levels with a power of 0.80 and a significance level of .05. To account for potential dropouts, a total of 50 patients were recruited. Materials and methods flowchart are presented in Fig. 1.

## 3. Results

The demographic characteristics of the study participants are shown in Table 1. The sample included 50 patients diagnosed with AV, with a mean age of 22.62 years (±3.98). This relatively narrow SD suggests a homogeneous age distribution across the cohort. Gender distribution indicated a predominance of female participants, with 35 females (70%) and 15 males (30%), resulting in a 3:1 female-to-male ratio. Although this gender imbalance is noted, further analysis regarding its potential implications on thyroid function outcomes was not conducted. The age range and gender ratio align with the demographic profile of individuals most commonly affected by AV.

**Table 1:** Demographic characteristics of study participants.

	N	Minimum	Maximum	Mean	Standard deviation
Age in year	50	18	35	22.62	3.98
Gender (n = 50)		Male		Female	
No.		15		35	
%		30		70	

Table 2 summarizes the thyroid function test results, including serum levels of TSH, T3, and T4 before and after IOS treatment. At baseline, the mean TSH level was 2.21 ± 0.88 uIU/mL, while the posttreatment mean increased significantly to 4.10 ± 1.55 uIU/mL (*P* < .00001). This marked elevation in TSH suggests a potential hypothyroid-like response to IOS therapy, although posttreatment levels remained within the upper limit of the normal reference range (0.30–4.20 uIU/mL). The SD for posttreatment TSH levels indicates greater variability, which may reflect individual differences in drug metabolism or treatment adherence.

**Table 2:** The values of thyroid function tests, in isotretinoin-treated patients (n = 50) at baseline and after treatment.

	Mean	±SD	*P*
Initial TSH	2.21	.88	.000
Follow up TSH	4.10	1.55	
Initial T4	15.99	2.38	.0001
Follow up T4	14.21	2.08	
Initial T3	4.62	.63	.000
Follow up T3	4.22	.63	

SD = standard deviation, T3 = triiodothyronine, T4 = thyroxine, TSH = thyroid-stimulating hormone.

Conversely, serum T4 levels showed a statistically significant decline from a baseline mean of 15.99 ± 2.38 pmol/L to 14.21 ± 2.08 pmol/L posttreatment (*P* < .0001). Similarly, mean T3 levels decreased from 4.62 ± 0.63 pmol/L at baseline to 4.22 ± 0.63 pmol/L following treatment (*P* < .0001). Both reductions remained within the normal physiological ranges (T4: 12.0–22.0 pmol/L, T3: 3.10–6.80 pmol/L). These findings indicate that IOS treatment may induce subclinical thyroid function alterations, characterized by an increase in TSH and reductions in T3 and T4 levels.

**Figure 2.**
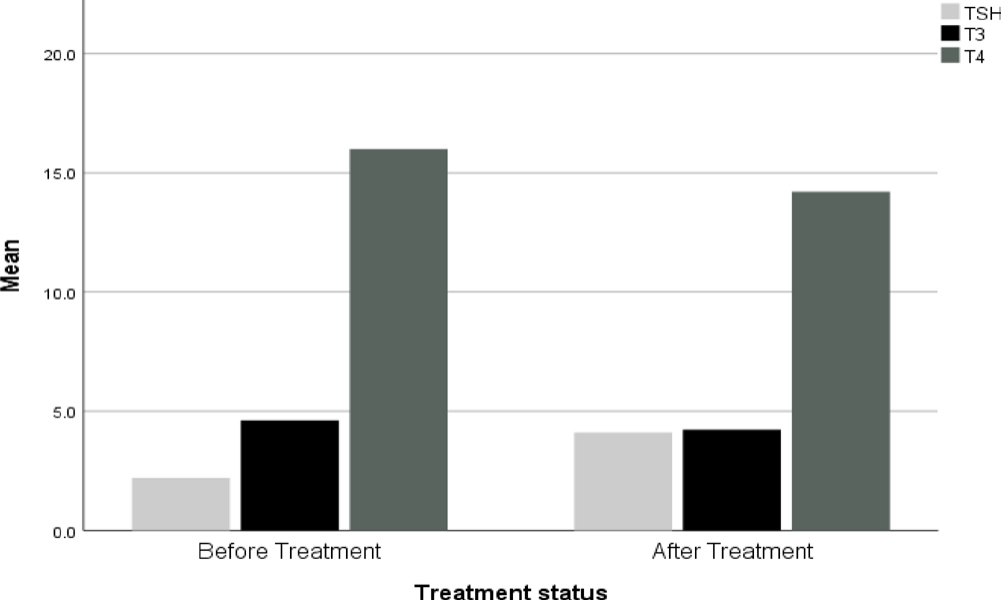
The levels of TSH, T3, and T4 pre- and post-IOS treatment. IOS = isotretinoin, T3 = triiodothyronine, T4 = thyroxine, TSH = thyroid-stimulating hormone.

Figure 2 illustrates the mean levels of TSH, T3, and T4 before and after IOS treatment. The graph clearly depicts an upward trend in TSH levels and downward trends in T3 and T4 levels. While these changes were statistically significant, their clinical significance warrants further investigation. Notably, none of the participants exhibited overt hypothyroidism, as defined by TSH levels exceeding the upper reference limit. However, the observed shifts may suggest a transient hypothyroid state or subclinical changes requiring monitoring.

The paired *t* test analysis confirmed the statistical significance of changes in thyroid parameters, with *P* values far below the .05 threshold. However, the absence of confidence intervals limits the interpretation of effect size and precision. Additionally, while the observed changes are statistically significant, their clinical impact remains uncertain. Whether these alterations in thyroid function require intervention or adjustment in treatment protocols remains an open question.

## 4. Discussion

This study confirms that IOS therapy in patients with AV is associated with statistically significant changes in thyroid function. Specifically, we observed elevated TSH levels and decreased T3 and T4 levels after a 4-month treatment period. These findings provide prospective evidence that IOS may influence the hypothalamic–pituitary–thyroid axis and support emerging concerns about its systemic endocrine effects.

The demographic characteristics of our sample (mean age 22.62 years, 70% female) are consistent with prior studies involving AV patients undergoing IOS therapy, such as Yildirim et al.^[15]^ The relatively homogeneous population helps to isolate the drug’s potential impact on thyroid function.

Several mechanisms have been proposed to explain the interaction between retinoids and thyroid hormones. IOS, through its action on retinoic acid receptors and Retinoid X Receptors, can modulate nuclear hormone receptors that influence TSH production and thyroid hormone metabolism.^[16–19]^ In previous studies, IOS has been associated with reduced thyroid volume and follicular cell apoptosis,^[20,21]^ potentially contributing to the decline in T3 and T4 levels observed in this cohort.

Our results align with earlier findings by Marsden et al^[22]^ and Karadag et al,^[23]^ who reported reductions in circulating thyroid hormone levels during IOS therapy. However, O’Leary et al^[24]^ observed no significant changes, reflecting inconsistency in the literature. These differences may be due to variability in sample sizes, treatment durations, and hormonal measurement techniques.

Notably, while posttreatment TSH levels increased significantly, they remained within the normal reference range for most patients. No participants developed overt hypothyroidism, indicating that changes may represent subclinical or transient thyroid dysfunction. The presence of such subclinical shifts supports the call for routine thyroid monitoring during long-term IOS use.

The predominance of female participants (70%) in this study reflects the higher prevalence of AV in young women seeking treatment. However, the potential influence of gender on thyroid function outcomes with IOS treatment remains unclear and warrants further investigation. Hormonal variations between genders may affect thyroid function and responsiveness to systemic therapies like IOS.^[13,15,25]^

Limitations such as the absence of a control group, non-standardized timing of blood sampling (which may influence TSH due to circadian rhythms), and lack of free T3/T4 measurements should be noted. These factors could impact the precision and clinical relevance of our findings. Moreover, the predominance of female participants raises the possibility of sex hormone interactions, which were not accounted for.

What is novel about our study is its prospective design with paired hormonal assessments, consistent dosing (0.5–1 mg/kg), and 4-month duration, reflective of routine clinical practice. By demonstrating consistent and statistically robust endocrine shifts post-IOS therapy, our findings reinforce the need for risk-stratified monitoring protocols.

These findings underscore the need for clinicians to monitor thyroid function in patients undergoing IOS treatment, particularly over extended periods. Routine TFTs could help identify subclinical hypothyroidism early, potentially mitigating adverse effects. This study highlights the importance of personalized treatment strategies, especially for patients with preexisting thyroid disorders or those at higher risk of hypothyroidism.

## 5. Recommendations for future research

Future research should prioritize investigating the long-term impact of IOS on thyroid function in larger, more diverse populations. Randomized controlled trials could provide more robust evidence, while mechanistic studies could explore IOS’s interactions with thyroid receptors and its apoptotic effects on thyroid cells. Additionally, exploring the role of gender differences in IOS-induced thyroid dysfunction may yield valuable insights into personalized treatment approaches. Extended follow-up periods, exceeding 6 months or even a year, could help determine whether observed changes in thyroid parameters are transient or persistent. Incorporating detailed imaging and functional thyroid assessments will enhance the understanding of IOS’s effects and support the development of safer treatment protocols. Investigating potential genetic predispositions to thyroid dysfunction in IOS-treated patients may also enhance understanding of individual variability in treatment responses.

## 6. Conclusion

IOS treatment in AV patients appears to induce subclinical thyroid function changes. Until larger controlled studies can confirm these associations, clinicians should consider baseline and periodic TFTs, particularly in patients with preexisting thyroid disorders or symptoms suggestive of hypothyroidism. Personalized treatment approaches, including endocrinology consultation when necessary, may improve overall safety and outcomes for patients undergoing systemic retinoid therapy.

## Acknowledgments

The authors extend their appreciation to Prince Sattam Bin Abdulaziz University for funding this research work through the project number (PSAU/2023/03/2725).

## Author contributions

**Conceptualization:** Mamdouh Ali Kotb.

**Data curation:** Mamdouh Ali Kotb, Othman Abahussein.

**Formal analysis:** Ramadan S. Hussein, Salman Bin Dayel.

**Investigation:** Mamdouh Ali Kotb.

**Methodology:** Ramadan S. Hussein.

**Supervision:** Ramadan S. Hussein.

**Validation:** Othman Abahussein.

**Visualization:** Salman Bin Dayel.

**Writing – original draft:** Ramadan S. Hussein.

**Writing – review & editing:** Ramadan S. Hussein, Salman Bin Dayel, Othman Abahussein.
